# Targeting Catecholaminergic Systems in Transgenic Rats With a CAV-2 Vector Harboring a Cre-Dependent DREADD Cassette

**DOI:** 10.3389/fnmol.2020.00121

**Published:** 2020-07-03

**Authors:** Juan-Carlos Cerpa, Alain R. Marchand, Yoan Salafranque, Jean-Rémi Pape, Eric J. Kremer, Etienne Coutureau

**Affiliations:** ^1^CNRS, Institut de Neurosciences Cognitives et Intégratives d’Aquitaine, Bordeaux, France; ^2^Institut de Neurosciences Cognitives et Intégratives d’Aquitaine, Université de Bordeaux, Bordeaux, France; ^3^Institut de Génétique Moléculaire de Montpellier, University of Montpellier, CNRS, Montpellier, France

**Keywords:** DREADD, CAV-2, orbitofrontal cortex, striatum, dopamine, noradrenaline

## Abstract

Techniques that allow the manipulation of specific neural circuits have greatly increased in the past few years. DREADDs (Designer receptors exclusively activated by designer drugs) provide an elegant way to manipulate individual brain structures and/or neural circuits, including neuromodulatory pathways. Considerable efforts have been made to increase cell-type specificity of DREADD expression while decreasing possible limitations due to multiple viral vectors injections. In line with this, a retrograde canine adenovirus type 2 (CAV-2) vector carrying a Cre-dependent DREADD cassette has been recently developed. In combination with Cre-driver transgenic animals, the vector allows one to target neuromodulatory pathways with cell-type specificity. In the present study, we specifically targeted catecholaminergic pathways by injecting the vector in knock-in rat line containing Cre recombinase cassette under the control of the tyrosine hydroxylase promoter. We assessed the efficacy of infection of the nigrostriatal pathway and the catecholaminergic pathways ascending to the orbitofrontal cortex (OFC) and found cell-type-specific DREADD expression.

## Introduction

Elucidation of neural circuits underlying complex animal behaviors depends on tools allowing control of neuronal activity. Designer receptors exclusively activated by designer drugs (DREADDs) technology (Roth, 2016) has been extensively used to transiently modulate activity within brain systems (Smith et al., 2016; Parkes et al., 2018). Intersectional approaches permit refined chemogenetic manipulation of specific circuits by targeting projections-defined neurons. Indeed, combining the injection of a canine adenovirus type 2 (CAV-2) vector carrying a Cre recombinase expression cassette, in the target region of a pathway, with the injection of Cre-dependent DREADDs, in the region of origin, provides a way to investigate the impact of activation (or inhibition) of specific pathways between brain areas (Alcaraz et al., 2018).

One of the main drawbacks of such a strategy is that it requires two vectors, which obviously increases the likelihood of injections misplacements and also raises the problem of the proportion of infected cells. To overcome these limitations, we report here a strategy that combines a single CAV-2 vector carrying a Cre-dependent DREADD cassette (CAV-hM4Di) and a Cre driver rat line. We assessed the efficacy of this method in the nigrostriatal dopaminergic pathway reaching the dorsolateral striatum (DLS), and the catecholaminergic pathways reaching the orbitofrontal cortex (OFC). We injected CAV-hM4Di in either the DLS or the OFC of TH-cre rats which express Cre-recombinase in dopaminergic and noradrenergic neurons. We show that this new CAV-2-based viral vector can specifically and effectively transduce catecholaminergic neurons with both projection and neuronal specificity.

## Materials and Methods

### Animals and Housing Conditions

Animals were six male heterozygous TH-cre+ rats on a Long-Evans background [Long Evans-Tg(TH-cre)3.1Deis] as well as two male transgene-negative TH-cre^−^ littermates, bred in our laboratory, and one male Long Evans rat (wild-type) obtained from Centre d’Elevage Janvier (France). Rats were aged 4 months at the time of the experiment and housed in pairs with *ad libitum* access to food and water. The facility was maintained at 21 ± 1°C with lights on from 08:00 to 20:00. Environmental enrichment was provided by orange-tinted polycarbonate tubing elements, following current French (Council directive 2013-118, February 1, 2013) and European (directive 2010-63, September 22, 2010, European Community) laws and policies regarding animal experiments.

### Viral Vector

An E1/E3-deleted, replication-defective, CAV-2 vector carrying double-inverted flox sites flanking a hM4Di-mCherry fusion protein expression cassette (CAV-DIO-hM4Di-mCherry, concentration 1 × 10^12^ particles/ml) was obtained from Biocampus PVM, Montpellier, France. The vector will be hereafter mentioned as CAV-hM4Di.

### Surgery

Rats were anesthetized with 5% isoflurane and placed in a stereotaxic frame with atraumatic ear bars (Kopf Instruments) in a flat skull position in which Bregma and Lambda are located at the same mediolateral and dorsoventral coordinates. Anesthesia was maintained with 1.5% isoflurane and complemented with a subcutaneous injection of analgesic ropivacaïne (a bolus of 0.1 ml at 2 mg/ml) at the incision locus. Intracerebral injections were made with a pump (UMP3-1 and Micro4 Controller, World Precision Instruments) *via* a 10 μl NanoFil syringe with a blunt, 34G needle. In the case of TH-Cre^+^ rats, 1 μl of CAV-hM4Di was injected unilaterally at two sites of the OFC. Coordinates were: +4.2 anteroposterior (AP); ± 1.6 medio-lateral (ML); −5 dorso-ventral (DV) and +3.2 AP; ± 2.4 ML; −5.6 DV. For DLS, 1 μl of CAV-hM4Di was injected unilaterally at one site. Coordinates were: +0.7 AP; ± 3.6 ML; −5 DV. In the case of transgene-negative littermate TH-cre^−^ and wild-type rats, 1 μl of CAV-hM4Di was injected unilaterally at one site of the OFC. Coordinates were +3.5 AP; +2.2 ML; −5.4 DV. All coordinates are given in millimeters from Bregma (Paxinos and Watson, 2014). The infusion was made at a rate of 200 nl/min and the pipette was left in place for an additional 5 min to allow diffusion of CAV-hM4Di. During recovery, rats were monitored and weighed daily. For an optimal migration and expression of the hM4Di-mCherry, we waited 4 weeks before perfusion.

### Immunohistochemistry

Rats were rapidly and deeply anesthetized with an overdose of sodium pentobarbital (Exagon^®^ Euthasol) and perfused transcardially with 60 ml of saline followed by 260 ml of 4% paraformaldehyde (4% PFA) in 0.1 M phosphate buffer (PB). Brains were removed and postfixed in the same 4% PFA solution overnight and then transferred to a 0.1 M PB solution. Subsequently, 40-μm-thick coronal sections were cut using a VT1200S Vibratome (Leica Microsystems). To form a series, every fourth section was collected into a cryoprotective solution and stored at −20°C. Fluorescent immunoreactivity was performed for mCherry and tyrosine hydroxylase (TH). Free-floating sections were first rinsed in 0.1 M PB saline (0.1 M PBS; 4 × 5 min) and then incubated in a blocking solution (0.1 M PBS, 0.3% Triton X-100, 3% of goat serum) for 1 h. Sections were then incubated with both primary antibodies rabbit anti-RFP (1/1,000 in blocking solution, PM005 MBL International Corporation) and monoclonal mouse anti-TH (1/2,500 in blocking solution, MAB318 Merck Millipore), for 48 h at 4°C on a shaker. After further rinses in 0.1 M PBS (4 × 5 min), sections were placed for 2 h in a bath containing both secondary antibodies TRITC goat anti-rabbit (1/200 in 0.1 M PBS, Jackson ImmunoResearch, code 111-025-003) and FITC goat anti-mouse (1/200 in 0.1 M PBS, Jackson ImmunoResearch, code 115-095-003) for 90 min on a shaker at room temperature. Following rinses in 0.1 M PBS (4 × 5 min), they were then incubated with Hoescht solution for counterstaining (1/5,000 in 0.1 M PBS, bisBenzimide H 33258, Sigma Aldrich, B2883) for 15 min on a shaker at room temperature. Finally, sections were rinsed with 0.1 M PBS, mounted in 0.05 M PB onto gelatin-coated slides, and coverslipped with Fluoromount G (SouthernBiotech, 0100-01). Sections were then imaged using an epifluorescence microscope (Olympus IX81) equipped with a camera (Orca ER, Hamamatsu) controlled by MicroManager Software (MM Studio).

For visible immunostaining, sections were rinsed in 0.1 M PBS containing 0.3% Triton X-100 (PBST; 4 × 5 min) and then incubated in PBST containing 1% hydrogen peroxide solution (H_2_O_2_) for 30 min. Further rinses were performed using PBST (4 × 5 min) before incubation in blocking solution for 1 h at room temperature. Then, sections were incubated in primary antibody rabbit anti-RFP (1/2,500 in blocking solution, PM005 MBL International Corporation) for 48 h at 4°C on a shaker. After further rinses in 0.1 M PBS (4 × 5 min), sections were placed for 2 h in a bath containing biotinylated goat anti-rabbit secondary antibody (1/1,000 in 0.1 M PBS, 111-065-003 Jackson ImmunoResearch) for 90 min at room temperature. Following rinses in 0.1 M PBS (4 × 5 min), they were then incubated with avidin-biotin-peroxidase complex (1/200 in 0.1 M PBS, 32020 ThermoFisher Scientific) for 90 min at room temperature. The final staining was made with diaminobenzidine (DAB, 10 mg tablet, D5905 Sigma-Aldrich) dissolved in 50 ml of 0.05 M Tris buffer (0.05 M TB) and 30 μl H_2_O_2_. Sections were then rinsed in 0.05 M TB (2 × 5 min) and then 0.05 M PB (2 × 5 min). They were collected on gelatin-coated slides, dehydrated with xylene, mounted and coverslipped using Eukitt mounting medium. Sections were scanned using a Nanozoomer slide scanner (Hamamatsu Photonics) with a 20× lens and analyzed with the NDP.view 2 freeware (Hamamatsu Photonics).

## Results

### Targeting the Dopaminergic Pathway Reaching the Dorsolateral Striatum

**Figure 1:**
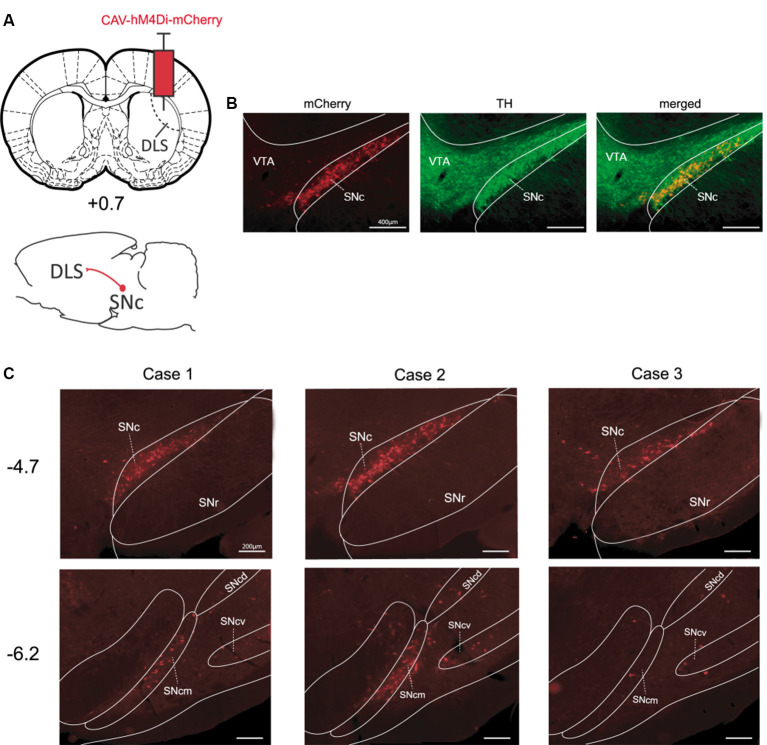
Targeting the nigrostriatal pathway with CAV-hM4Di **(A)** Schematic illustration of viral vector injection into the dorsolateral striatum (DLS) to selectively infect nigrostriatal neurons. **(B)** Example of staining obtained in the SNc vs. the VTA for mCherry-positive and tyrosine hydroxylase (TH)-positive neurons [Case 2, anteroposterior (AP) −4.7 mm]. **(C)** The rostrocaudal extent of staining in SN for all pilot cases at coordinates AP −4.7 mm and AP −6.2 mm (from Bregma). DLS, dorsolateral striatum; SNc, substantia nigra pars compacta; SNcm, d, v: substantia nigra pars compacta, medial tier, dorsal tier, and ventral tier respectively; VTA, ventral tegmental area.

It has extensively been shown that the substantia nigra pars compacta (SNc) sends major projections to the dorsal striatum (Fallon and Moore, 1978). To selectively target this nigrostriatal pathway we injected CAV-hM4Di into the DLS of TH-cre^+^ rats (Figure 1A). As expected, this manipulation resulted in a strong expression of hM4Di-mCherry mainly in cell bodies located unilaterally in the ipsilateral SNc, nevertheless, fluorescence can also be observed in axons originating from these cell bodies (Figure 1B). Moreover, the localization of these stained cell bodies corresponded to the localization of TH-positive cells as shown in Figure 1B, suggesting that neurons expressing hM4Di-mCherry are primarily dopaminergic cells. In these examples, we found that approximately 38% (417/1091) of TH-positive cells in the ipsilateral SNc expressed mCherry. The same Figure shows that dopaminergic cells in the ventral tegmental area (VTA), which do not target the DLS, did not express hM4Di-mCherry. Consistent with the latter, we did not observe labeled cells elsewhere in the brain. Interestingly, after a single injection into the DLS, the staining of cells covered a substantial distance along the rostrocaudal axis of the SNc (Figure 1C). Indeed, strong hM4Di-mCherry labeling of cells was found at AP −4.7 mm (from Bregma) in all cases, and numerous hM4Di-mCherry positive cells were found even more posteriorly at AP −6.2 mm (from Bregma) in at two out of three cases.

### Targeting Catecholaminergic Pathways Ascending Into the Orbitofrontal Cortex

**Figure 2:**
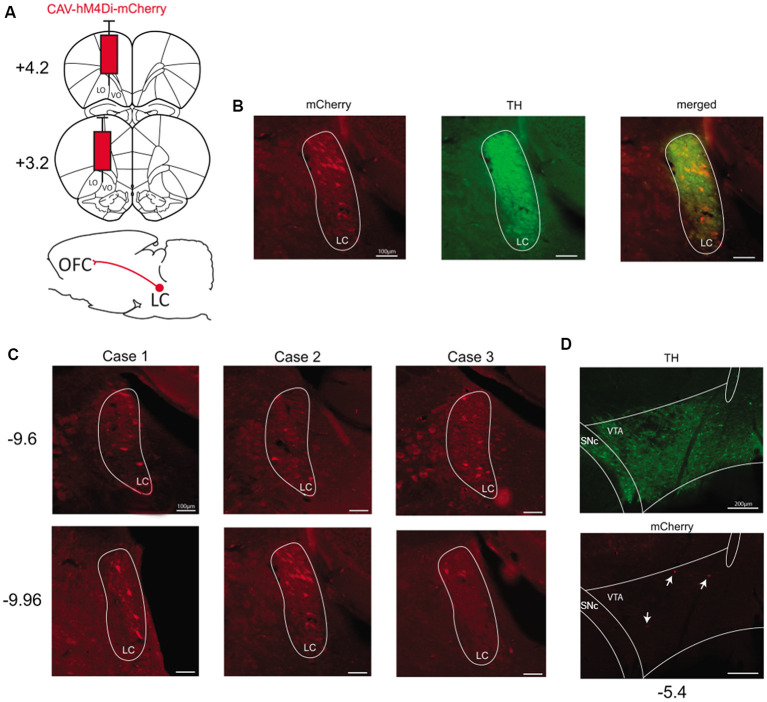
Targeting the catecholaminergic pathway to the OFC. **(A)** Schematic illustration of viral vector injection into the OFC at two different coordinates taken from Bregma (AP +3.2 mm and AP +4.2 mm). **(B)** Example of staining obtained in the LC for mCherry-positive and TH-positive neurons (Case 2, AP −9.96 mm). **(C)** Staining obtained in LC for all pilot cases at two different anteroposterior coordinates taken from Bregma (AP −9.6 mm and AP −9.96 mm). **(D)** Example of staining obtained in the VTA for mCherry-positive and TH-positive neurons (AP −5.4 mm). OFC, orbitofrontal cortex; VO, ventral OFC; LO, lateral OFC; LC, locus coeruleus; VTA, ventral tegmental area.

To express hM4Di-mCherry in catecholaminergic pathways reaching the OFC, we injected CAV-hM4Di in the ventral and lateral portions of OFC (Figure 2A). We found substantial labeled neurons unilaterally in the ipsilateral locus coeruleus (LC). The localization of stained cells corresponded to TH-positive cells which are noradrenergic in the LC (Figure 2B). In all cases, we observed mCherry staining along the central portion of LC. Indeed, as observed in Figure 2C, stained cells were mostly located from AP −9.6 mm to −9.96 mm (from Bregma). However, regarding the dopaminergic pathway reaching the OFC, we found no staining or very few stained cells in the ipsilateral VTA (Figure 2D). Again, there was no evidence of labeled cells elsewhere in the brain, suggesting that the floxed hM4Di-mCherry cassette was inverted selectively in TH-positive and Cre-expressing cells.

**Figure 3:**
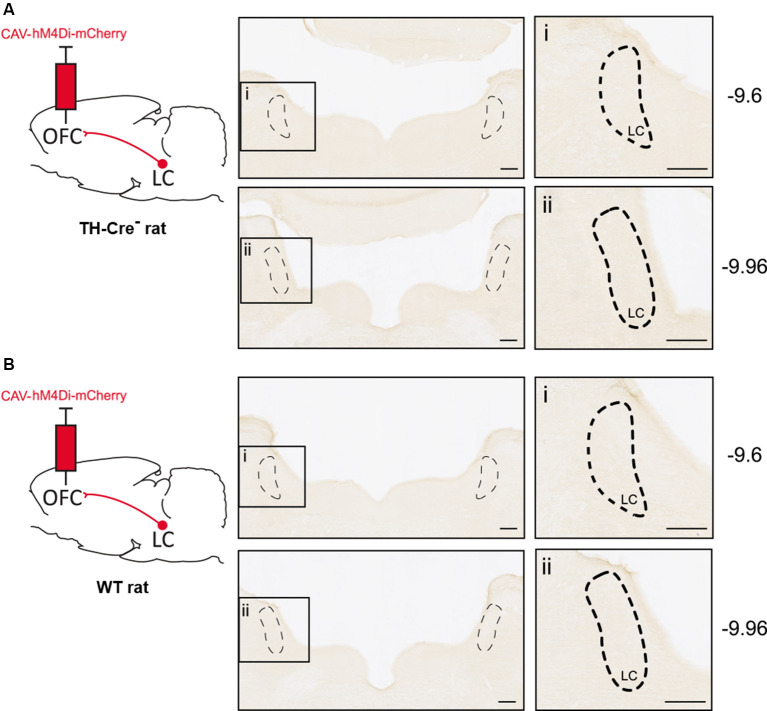
Absence of CAV-hM4Di expression in animals lacking Cre-recombinase. **(A)** DAB immunostaining in LC after injection of CAV-hM4Di in the OFC of transgene-negative littermate TH-cre^−^ at two AP coordinates (AP −9.6 mm and AP −9.96 mm). **(B)** DAB immunostaining in LC with the same injection in wild-type rats.

Furthermore, we found no staining in LC after injection of CAV-hM4Di in the OFC of transgene-negative littermates TH-Cre- (one case reported in Figure 3A) or a wild-type animal (Figure 3B). Thus, in the absence of Cre-recombinase, no expression of DREADD is observable.

## Discussion

Our findings demonstrate that CAV-hM4Di can be used to express hM4Di-mCherry in different pathways using a single injection in a transgenic rat line. Indeed, injection of this vector in two different structures, specifically the DLS and the OFC, resulted in a reliable mCherry-reporter staining in catecholaminergic nuclei projecting to these structures.

Numerous studies have used DREADD technology to study the function of dopaminergic pathways using a combination of viral vectors and transgenic rodent lines (Runegaard et al., 2019). Our report provides evidence to consider CAV-hM4Di as a new powerful tool to transiently inhibit the nigrostriatal dopaminergic pathway when used in combination with the TH-transgenic rodent line. Indeed, a single injection of CAV-hM4Di in DLS primarily resulted in strong staining at the level of the SNc along the rostrocaudal axis. Such staining is not surprising due to the strong connectivity between these two structures as previously described (Fallon and Moore, 1978). The neighboring VTA was not stained, confirming the anatomic specificity of the infection.

The pattern of expression we obtained in the nigrostriatal pathway looks very similar to both qualitatively and quantitatively to other reports reporting behavioral effects (Boekhoudt et al., 2016; Bouchet et al., 2018). The efficiency of expression in Bouchet et al. (2018) is not quantified. However, Boekhoudt et al. (2016), using a double-viral strategy to target the nigrostriatal pathway, have reported efficiency of 34.8% of SN (dopaminergic) neurons expressing the fluorescent reporter mCherry. In the three cases shown here, expression of mCherry was found in 38% of TH-positive neurons. Therefore, our strategy seems to be as effective as that of previous reports.

It is also important to note that a behavioral effect can be found with a very small proportion of neurons expressing the viral construct (~3% in dlPFC of monkeys, Upright et al., 2018).

Recent studies have highlighted the involvement of noradrenergic pathways reaching different prefrontal areas to support distinct cognitive functions (Tervo et al., 2014; Cope et al., 2019). Therefore, simple methods allowing transient and specific modulation of these pathways are necessary to understand their functional specificity. In this study, we have provided an example of such a possibility by targeting the noradrenergic inputs into the OFC. The brainstem nucleus, LC, constitutes the major source of noradrenaline (NA) to the cortex (Loughlin et al., 1982) and sends projections to all prefrontal areas (Agster et al., 2013; Chandler et al., 2013; Cerpa et al., 2019). Accordingly, following CAV-hM4Di injection in the OFC, we found staining restricted to the LC. Furthermore, in all cases, labeled cells were mostly found in the medial portion of the LC. This is in agreement with the topographic organization of LC efferent neurons showing that, along the rostrocaudal axis, neurons projecting to the neocortex are primarily located in the medial portion of LC (Loughlin et al., 1986).

In the present study, OFC injections only resulted in weak and rare staining in the VTA. Considering that the rodent OFC dopaminergic innervation exists (Chandler et al., 2013) but is only scarce (Descarries et al., 1987; Murphy and Deutch, 2018), it is difficult to conclude on the efficiency of the vector to infect this pathway. However, because the infection of neurons by CAV-2 vectors depends on CAR availability (Bru et al., 2010), one possibility is that mesocortical neurons reaching the OFC express few CAR molecules. Interestingly, recent studies have used a “CAR boost” strategy to enhance CAV-2 tropism in various pathways (Li et al., 2018). Such complementation could be used to promote CAV-hM4Di infection in pathways of interest.

The present report highlights the use of CAV-2 vector harboring a DREADD cassette with TH-Cre rats. However, it does not give an exhaustive presentation of other possibilities of single intracerebral injections. Indeed, while a similar study in TH-Cre mice is lacking, it is likely that CAV-hM4Di could be used in mice and in combination with other types of transgenic lines e.g Dbh-Cre mice, SERT-Cre mice. Moreover, CAV-2 vectors have the potential to include an expression cassette as large as 30 kb. Therefore, our proof-of-principle studies are a step towards using vectors with multiple expression cassettes. For example, one could imagine an excitatory DREADD, an inhibitory DREADD, and a drug inducible reporter gene cassette in the same vector. In addition, CAV-hM4Di could still be used in wild type animals with Cre provided by another viral vector (e.g AAV-cre vector) that infects neurons at the site of injection.

In control animals, lacking the Cre-recombinase, we found no evidence of recombination and expression of the hM4Di-mCherry indicating specific Cre-dependent expression. On the other hand, it has been argued that some non-specific expression could be observed with the use of floxed AAVs, as stated in this issue (Morceau et al., 2019). The present study suggests that the CAV-2 vector containing a floxed DREADD could be used to overcome this issue.

## Data Availability Statement

The raw data supporting the conclusions of this article will be made available by the authors, without undue reservation, to any qualified researcher.

## Ethics Statement

The animal study was reviewed and approved by Bordeaux Ethics Committee (Université de Bordeaux).

## Author Contributions

J-CC, J-RP, YS, and EC performed research. J-CC, AM, EK, and EC wrote and edited the article.

## Conflict of Interest

The authors declare that the research was conducted in the absence of any commercial or financial relationships that could be construed as a potential conflict of interest.
